# Impact of TP53 mutations in Triple Negative Breast Cancer

**DOI:** 10.1038/s41698-022-00303-6

**Published:** 2022-09-09

**Authors:** Zahi I. Mitri, Nour Abuhadra, Shaun M. Goodyear, Evthokia A. Hobbs, Andy Kaempf, Alastair M. Thompson, Stacy L. Moulder

**Affiliations:** 1 Knight Cancer Institute, Oregon Health & Science University, Portland, OR USA; 2 Memorial Sloan Kettering Cancer Center, New York City, NY USA; 3 Baylor College of Medicine, Houston, TX USA; 4 Eli Lilly and Company, Indianapolis, IN USA

**Keywords:** Breast cancer, Prognostic markers

## Abstract

Identifying triple negative breast cancer (TNBC) patients expected to have poor outcomes provides an opportunity to enhance clinical management. We applied an Evolutionary Action Score to functionally characterize *TP53* mutations (EAp53) in 96 TNBC patients and observed that EAp53 stratification may identify *TP53* mutations associated with worse outcomes. These findings merit further exploration in larger TNBC cohorts and in patients treated with neoadjuvant chemotherapy regimens.

## Introduction

Triple Negative Breast Cancer (TNBC) has typically been treated with anthracycline-based neoadjuvant chemotherapy (NACT), but about half such patients fail to achieve a pathologic compete response (pCR) at the time of surgery^1–4^. The high prevalence (60%) of *TP53* mutations (*TP53mut*) in TNBC makes it an attractive therapeutic target and marker to predict chemotherapy sensitivity^5–7^; however, the functional significance of individual *TP53mut* remain unclear^8–13^. Mutational diversity suggests that not all *TP53mut* are equivalent and that alterations to specific protein domains will have differential effects on the gain-of-function oncogenic activity, including chemotherapy resistance^9,14–17^. The computational Evolutionary Action Score of *TP53* coding variants (EAp53) offers an alternative approach to functionally characterizing *TP53mut* and predicting NACT response^15,18,19^. EAp53 incorporates evolutionary variations to predict the impact of somatic mutations on likelihood of amino acid substitution and p53 protein function^20^. We report on the identity and prognostic value of *TP53mut* and EAp53 in a single institution cohort of patients with primary TNBC.

**Fig. 1:**
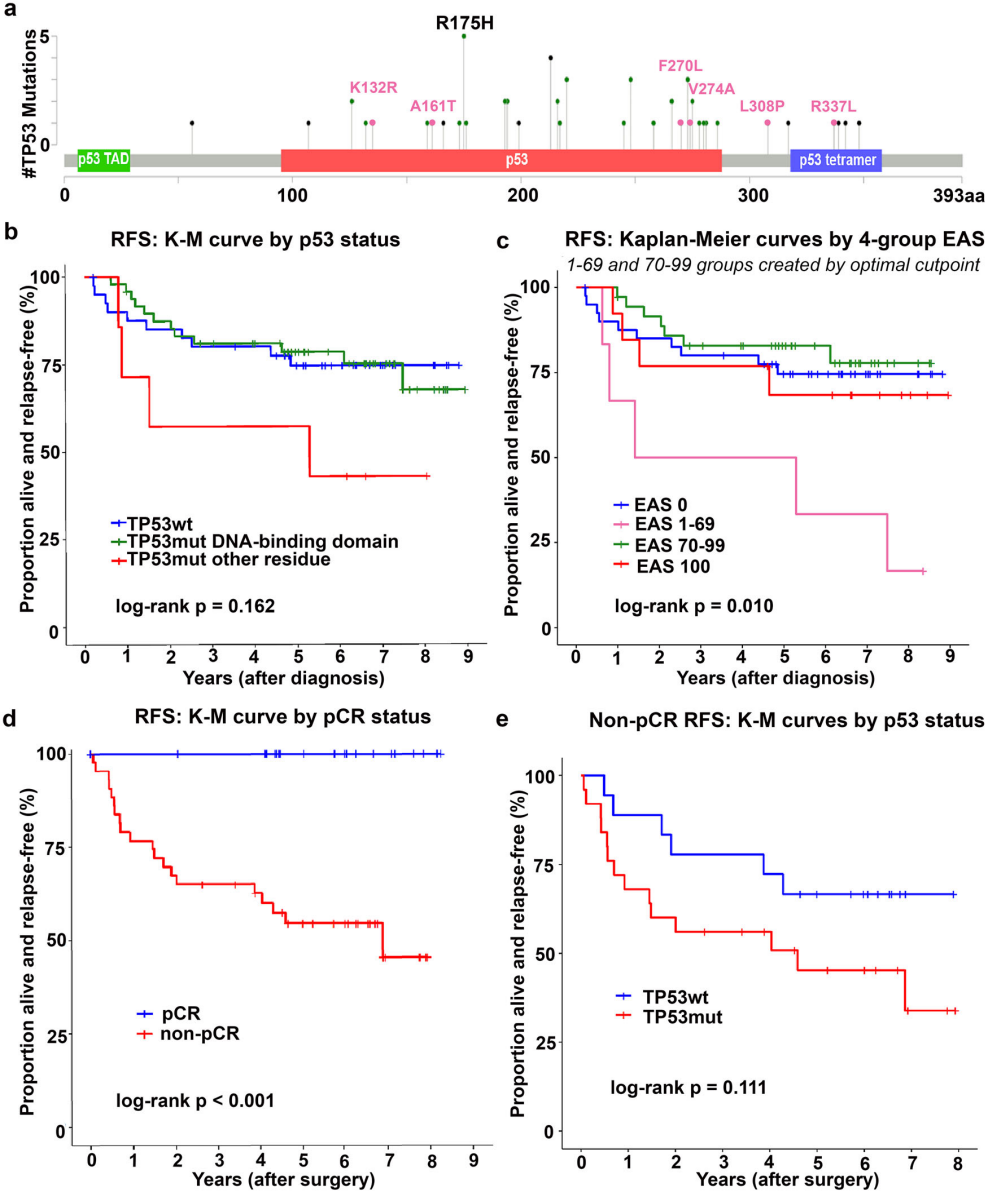
Association of TP53 mutational status and clinical outcomes. **a** Lollipop plot showing location and sequence of TP53 mutations in study cohort. Pink mutations denote those comprising the EAp53 1-69 group. **b** RFS Kaplan–Meier curves by tumor TP53 mutation status and location (log-rank test *p* = 0.162). **c** Kaplan–Meier curves by EAp53 category. **d** RFS Kaplan–Meier curves by pCR status (log-rank test *p* < 0.001). **e** RFS Kaplan–Meier curves by tumor TP53 mutation status for 43 patients who did not achieve a pCR (log-rank test *p* = 0.111).

Among 96 patients enrolled in this study (MDACC Protocol # 2011-0007), 55 (57%) had a *TP53mut*, the majority (76%) of which were missense mutations (*TP53mis*; Fig. 1a, Supplemental Table 1). Table 1 summarizes clinical characteristics by *TP53mut* status. Median follow-up was 6 years from diagnosis, with 21 observed deaths (22% of patients); 5-year overall survival (OS) rate was 82% and median OS was not reached. Nineteen patients (20%) had a documented local or distant recurrence of disease, and the 3-year recurrence-free survival (RFS) rate was 79%. Patients with *TP53mut* tumors had slightly lower RFS compared to those with *TP53wt* tumors at 77.9% (95%CI 64.4–86.8) vs. 80.0% (95%CI 64.4%–89.5%) at 3 years, respectively, and HR of 1.15 (95%CI 0.52–2.54, *p* = 0.726). The inferior OS in *TP53mut* compared to *TP53wt* patients (81.3% [95%CI 68.0–89.5] vs. 90.0% [95%CI 75.5%–96.1%]; HR 1.54 [95%CI 0.62–3.82], *p* = 0.351) was also non-significant.

**Table 1:** Clinicopathological characteristics by p53 mutational status.

Clinical Characteristics	TP53 wild type (*n* = 41), N (%) or Value	TP53 mutant (*n* = 55), *N* (%) or Value	*p*
Age at diagnosis			
median (IQR)	56 (50–64)	55 (46–62)	0.147
range	30–83	21–79	
	0 *na*	0 *na*	
Age at diagnosis (binary)			
<50 years	10 (24%)	*n* = 20 (36%)	0.268
≥50 years	31 (76%)	*n* = 35 (64%)	
Race			
White	26 (63%)	33 (60%)	0.966
Black	7 (17%)	11 (20%)	
Hispanic	6 (15%)	7 (13%)	
Asian	2 (5%)	3 (5%)	
*na*		1 (2%)	
Laterality			
Left	19 (46%)	24 (44%)	0.838
Right	22 (54%)	31 (56%)	
Tumor volume (cm^3^)			
median (IQR)	15.1 (7.3–27.3)	14.9 (5.6–29.0)	0.950
range	0.6–837.8	0.8–594.0	
	2 *na*	1 *na*	
Clinical T stage			
T1	7 (17%)	10 (18%)	0.307
T2	31 (76%)	35 (64%)	
T3/T4	3 (7%)	10 (18%)	
Clinical N stage			
N0	23 (56%)	32 (58%)	0.921
N1	9 (22%)	13 (24%)	
N2/N3	9 (22%)	10 (18%)	
TP53 expression (TPM)			
median (IQR)	8.5 (4.5–17.9)	16.0 (8.9–23.5)	0.009
range	2.1–95.8	2.1–58.0	
EASp53 (RFS-optimal)			
0	41 (100%)	0 (0%)	<0.001
1–69	0 (0%)	6 (11%)	
70–99	0 (0%)	36 (65%)	
100	0 (0%)	13 (24%)	
Diagnosis to NACT, months			
Median (IQR)	1.4 (0.9–2.0)	1.2 (0.9–1.9)	0.715
range	0.1–7.7	0.5–4.6	
	12 <*na* >	10 <*na* >	
Taxane therapy			
No	11 (27%)	9 (16%)	0.310
Yes	30 (73%)	46 (84%)	
Anthracycline therapy			
No	14 (34%)	9 (16%)	0.055
Yes	27 (66%)	46 (84%)	
Platinum therapy			
No	39 (95%)	49 (89%)	0.460
Yes	2 (5%)	6 (11%)	
Diagnosis to surgery, months			
Median (IQR)	6.3 (1.9–7.5)	6.8 (5.3–7.6)	0.256
range	0.4–9.3	0.8–12.0	
	2 <*na* >	0 <*na* >	
Path T stage			
T0/Tis	9 (22%)	18 (33%)	0.113
T1	11 (27%)	19 (35%)	
T2	16 (39%)	10 (18%)	
T3	3 (7%)	8 (15%)	
*na*	2 (5%)	0 (0%)	
Path N stage			
N0	28 (68%)	39 (71%)	0.298
N1	10 (24%)	10 (18%)	
N2/N3	1 (2%)	6 (11%)	
*na*	(5%)	0 (0%)	
Path TNM stage			
0	8 (20%)	17 (31%)	0.012
I	8 (20%)	15 (27%)	
II	22 (54%)	14 (25%)	
III	1 (2%)	9 (16%)	
*na*	2 (5%)	0 (0%)	
pCR after NACT			
Yes	8 (20%)	17 (31%)	0.451
No	18 (44%)	25 (45%)	
*na*	15 (37%)	13 (24%)	

*IQR* interquartile range, *na* not available, *TPM* transcripts per million, *NACT* neoadjuvant chemotherapy, *pCR* pathological complete response.

Seven patients with *TP53mut* in a residue outside the DNA-binding domain (predominantly in the tetramerization domain, Fig. 1a) had markedly worse RFS (Fig. 1b; adjusted HR 3.17 [95%CI 0.98–10.22] vs. *TP53wt*, *p* = 0.054) and OS (Fig. 1a; adjusted HR 5.28 [95%CI 1.53–18.25] vs. *TP53wt*, *p* = 0.008), even when controlling for prognostic factors (i.e., age at diagnosis for RFS and clinical node positivity for RFS and OS). Survival estimates were similar in DNA-binding domain *TP53mut* and *TP53wt* patients (Fig. 1b, Supplemental Fig. 1A, B and Supplemental Table 2).

After applying the EAp53 algorithm^15,18,20–22^, patients were placed into one of four groups: *TP53wt* (*n* = 41) were assigned a score of 0 (i.e., EAp53 0), patients with a nonsense mutation (*TP53non*, *n* = 13) had a score of 100 (i.e., EAp53 100), and the remaining patients with missense mutations were divided into two groups that maximized the difference in RFS: EAp53 1-69 (*n* = 6; 4 patients with *TP53mut* in the DNA-binding domain) and EAp53 70-99 (*n* = 36; all patients with *TP53mut* in the DNA-binding domain). Patients with EAp53 1-69 had significantly worse RFS (50% [95%CI 11.1%–80.4%]; HR 4.17 [95%CI 1.41–12.31], *p* = 0.010) than those with EAp53 0 (80% [95%CI 64.0%–89.5%]) (Fig. 1c, Supplemental Fig. 1C). Controlling for age at TNBC diagnosis and clinical nodal status characteristics significantly correlated with recurrence or death, and did not dampen the effect of EAp53 1-69 on RFS (adjusted HR 3.90 [95%CI 1.30–11.67] vs. *TP53wt*, *p* = 0.015) (Supplemental Table 2). Additionally, patients with EAp53 1-69 had a higher risk of death (OS rate of 66.7% [95%CI 19.5%–90.4%]; HR 5.33 [95%CI 1.55–18.39], *p* = 0.008) compared to EAp53 0 patients (OS rate 90.0% [95%CI 75.5%–96.1%]) (Supplemental Table 2). This five-fold increase in the risk of death for EAp53 1-69 *TP53mis* compared to *TP53wt* patients persisted when controlling for clinical nodal status in the multivariable setting (adjusted HR 5.10 [95%CI 1.47–17.72], *p* = 0.010). There was little difference in RFS when comparing EAp53 0 to EAp53 70-99 (82.9% [95%CI 65.9%–91.9%]; HR 0.76 [95%CI 0.29–1.99], *p* = 0.571) and to EAp53 100 (76.9% [95%CI 44.2%–91.9%]; HR 1.18 [95%CI 0.37–3.78], *p* = 0.776) (Fig. 1c, Supplemental Table 2). As with RFS, OS for the EAp53 70-99 and EAp53 100 groups did not significantly differ from EAp53 0 (Supplemental Fig. 1C, Supplemental Table 2).

Among patients receiving NACT (*n* = 68), 25 (37%) achieved a pCR, and 43 (63%) had residual disease (Supplemental Table 3). Regardless of *TP53mut* status at TNBC diagnosis, patients achieving pCR after NACT had excellent outcomes, with 100% RFS at 3 years post-surgery (Fig. 1d). The lone significant predictor of pCR was clinical ≥N1 stage (odds ratio 0.30 [95%CI 0.10–0.83], *p* = 0.022); however, a noteworthy difference in pCR rates was observed between EAp53 groups, with 48% achieving pCR (12 of 25 patients) for EAp53 70-99, 45% (5 of 11) for EAp53 100, 31% pCR (8 of 26) for EAp53 0, and 0% pCR (0 of 6) for EAp53 1-69.

Among the 43 patients who failed to achieve a pCR after NACT, those with *TP53mut* tumors had inferior RFS (56.0% [95%CI 34.8%–72.7%]; HR 2.14 [95%CI 0.82–5.61], *p* = 0.120) compared to the *TP53wt* group (77.8% [95%CI 51.1%–91.0%]) (Fig. 1e, Supplemental Table 4). If the *TP53mut* occurred outside the protein’s DNA-binding domain (*n* = 4 non-pCR patients), RFS was significantly worse (25.0% [95%CI 0.9%–66.5%]; HR 6.97 [95%CI 1.93–25.10], *p* = 0.003) than *TP53wt*. Among non-responding patients, estimated RFS was longest for EAp53 0 (*TP53wt*), and shortest for EAp53 1-69 (50.0% [95%CI 11.1%–80.4%]; HR 3.31 [95%CI 1.00–11.02] vs. *TP53wt*, *p* = 0.051) and for EAS 100 (50.0% [95%CI 11.1%–80.4%]; HR 3.14 [95%CI 0.88–11.26] vs. *TP53wt*, *p* = 0.079) (Supplemental Table 4).

Understanding the functional impact of *TP53* mutations on treatment response and outcomes remains an elusive topic in cancer research and an area of active investigation^6,8,17,23–25^. In this study, the small group of patients harboring a missense mutation with EAp53 1-69 had the lowest pCR rate and shortest RFS and OS. The six specific mutations (listed in Supplemental Table 1) are uncommon in breast cancer, each occurring in <0.05% of cases according to the cBioPortal public data repository (24 studies comprising >10,000 cases, accessed 4-12-2022)^26,27^. Moreover, their functional significance remains largely unknown. The A161T mutation within the DNA-binding domain attenuates autophagy via mTOR and AMPK signaling, which is associated with resistance to anti-cancer therapy^28,29^. The F270 position within the DNA-binding domain is important for stabilizing the beta-sandwich structure of the p53 protein, and mutation to leucine is predicted to be a druggable target for small molecules to stabilize this type of p53 mutant^30–32^. Mutations at the R337 residue have a role in p53 methylation and ubiquitination, and R337L is thought to promote stability and sub-cellular localization of p53^33,34^. To our knowledge, the function of L308P, V274A, and K132R mutations have not been biochemically characterized^35,36^.

The majority (80%) of *TP53* mutations occur within the DNA-binding domain^37^. These mutations produce a defunct transcriptional product that is unable to respond to cellular stress,; resulting in mitotic catastrophe and apoptosis after chemotherapy that occurs independent of p53^38–40^. The transcriptional activity of p53 is largely dependent on tetramerization of individual p53 monomers. While mutations in the tetrameric region are less common (~20%), they can impact conformation of the DNA-binding domain, interaction with the transcriptional machinery, and p53 localization^41,42^. We observed that *TP53mut* occurring outside the DNA-binding domain (13% of *TP53mut* in our patient set) was associated with shorter RFS and OS compared to *TP53wt or TP53mut* in the DNA-binding domain. One of the mutations in the EAp53 1-69 group is also present within the tetrameric domain; however, the significance of any biological association remains to be determined, and further investigation may provide mechanistic insight into the poor outcomes observed in this study.

This single center study has several limitations, including a small sample size that led to wide confidence intervals and precluded us from validating our optimal EAp53 cut-point for missense mutations. Additionally, given the period during which our patients were treated, adjuvant capecitabine was not widely used for residual disease following NACT (non-pCR at the time of surgery), so the prognostic impact of adjuvant capecitabine, and its association with *TP53* mutation status, could not be captured. Similarly, use of pembrolizumab, which is becoming standards of care, could not be addressed here. Furthermore, we did not evaluate immune infiltrates or the presence of other genomic alterations that may impact efficacy of NACT and overall prognosis.

In this hypothesis-generating study, we identified two patient subsets (one defined by *TP53mut* location outside the DNA-binding domain and the other by *TP53mis* EAp53 1-69) demonstrating limited response to NACT and relatively high risk of disease recurrence and death. Our results also indicate that *TP53mut* corresponds with worse outcomes among patients with residual disease following NACT. These findings warrant further investigation in larger cohorts and within therapeutic clinical trials that cover a range of neoadjuvant regimens, in order to determine the functional impact of *TP53* mutations in TNBC; especially in light of promising therapies being developed to directly targeting *TP53mut* or indirectly targeting key components in the p53 signaling pathway^7,43–45^.

## Methods

### Patients

The TNBC patient cohort for this study consisted of 96 women treated at The University of Texas MD Anderson Cancer Center (MDACC) Breast Center between 2011 and 2016. The study was approved by the MDACC institutional review board (MDACC IRB#2011-0007). Written informed consent was obtained from all patients before enrollment. Patient demographics, tumor characteristics and treatment, surgical, pathological, and survival data were collected. *TP53* mutational status was determined through RNA sequencing using Nanodrop™ spectrophotometer (concentration, 260:280 OD ratio) and an Agilent Bioanalyzer (RNA integrity number [RIN] and RNA 28 S:18 S ratio).

### EAp53 Computation

Missense TP53 mutations were analyzed using the Evolutionary Action Scoring (EAS) System as previously described^15,18^. This Scoring System is based on the hypothesis that protein evolution is a continuous and differentiable process between genotype and phenotype. Therefore, if the phenotype (φ) is a function of the genotype (γ), *φ* = f (γ), then a change in phenotype (dφ) is equal to the scalar product of the gradient of the function (∇*f*) and the change in genotype (dγ). When considering a single mutation from amino acid X to any other amino acid Y at sequence position i, the genotype perturbation reduces to the magnitude of that substitution, denoted $\Delta r_{i,X \to Y}$, and is measured by the ranks of amino acid substitution odds^46^; however, these odds are computed for different deciles of the evolutionary gradient at the substituted position. The gradient (∇*f*) reduces to the partial derivative of the evolutionary fitness function for its *i*^th^ component, denoted ∂*f*/∂*r*_*i*_, and is measured by importance ranks of the Evolutionary Trace (ET) method^21,22^, according to which, residues that vary amongst closer homologous sequences are ranked less important than those that only vary amongst distant homologous sequences. This forms the equation: Δφ ≈ ∂*f*/·$\Delta {{{\mathrm{r}}}}_{{{{\mathrm{i}}}},{{{\mathrm{X}}}} \to {{{\mathrm{Y}}}}}$.

The result of this equation is normalized to create percentile scores for TP53 protein; for example, an EAS of 68 implies that the impact is >68% of all possible amino acid substitutions in p53. TP53 missense mutations were given EAp53 percentile scores, with higher scores representing alterations that are more impactful, according to ET theory. Wild-type TP53 sequences were scored as zero as this is the normally functioning protein and nonsense mutations were assigned a score of 100 to account for a truncated, non-functional protein.

### Statistical analysis

Patient, tumor, and treatment characteristics were summarized separately for *TP53wt* and *TP53mut* patients and compared across these groups using Fisher’s exact test (for categorical variables) or the Wilcoxon rank sum test (for continuous variables). Patients with a TP53 missense mutation (EAp53 1-99) were divided into 2 groups by applying an optimal cut-point that was identified as the EAp53 value that maximized the RFS log-rank test statistic for the resulting 2-group comparison. Therefore, creation of the EAp53 1-69 and 70-99 groups was a data-driven approach that (i) resulted in an RFS-optimized dichotomization of *TP53mis* patients and (ii) requires validation in an independent dataset. RFS and OS were measured from the date of TNBC diagnosis (if the predictor variables were known at this time) or the date of resection (e.g., for pCR status as the predictor), estimated with the Kaplan–Meier method, and compared with the log-rank test. All reported survival percentages pertain to 3 years post-diagnosis or post-surgery, depending on when the predictor variable was known. Cox proportional hazards regression models were fit to estimate hazard ratios (and compute Wald test p-values) that quantify the association between predictor variables and survival. An AIC-based backward elimination procedure was used to construct multivariable survival models from a set of predictors with univariable model *p*-values < 0.20. Associations between pre-treatment patient features and pCR were assessed with logistic regression. *P*-values < 0.05 were considered statistically significant and there was no adjustment for multiple tests (i.e., across univariable models and different outcomes). Analyses were performed and survival plots were created using R version 4.1.0.

### Reporting summary

Further information on research design is available in the Nature Research Reporting Summary linked to this article.

## Supplementary information

Reporting Summary

Informed Consent

Supplementary Material

## Data Availability

Consented data that can be released are included in this article and supplementary files, or are deposited in the SageBionetworks’ Synapse Platform (www.synapse.org) under accession syn33621227. Patients were not consented for the release of underlying sequence data and are not publicly available, but may be made available upon reasonable request from the corresponding author, Dr. Zahi Mitri. Specifically, access to individual patient-level data, for non-commercial purposes only, can only be shared per specific institutional review board (IRB) requirements. Upon reasonable request by each respective author/institution, a data sharing agreement may be initiated between the interested parties and the clinical institution following institution-specific guidelines.

## Code availability

Statistical analyses was conducted using R (version 4.1.0), and is available upon request. EAS was determined using a publically-available web-based tool: http://eaction.lichtargelab.org/.
