# Supplementary material for: Impact of TP53 mutations in Triple Negative Breast Cancer
Source: NPJ Precis Oncol. 2022 Sep 9;6:64. doi: 10.1038/s41698-022-00303-6 (PMC9463132; doi:10.1038/s41698-022-00303-6)
Supplement: Supplementary file 1 — Reporting Summary [file 41698_2022_303_MOESM1_ESM.pdf]

nature portfolio

Corresponding author(s): Zahi Mitri

Last updated by author(s): Apr 29, 2022

Reporting Summary

Nature Portfolio wishes to improve the reproducibility of the work that we publish. This form provides structure for consistency and transparency in reporting. For further information on Nature Portfolio policies, see our Editorial Policies and the Editorial Policy Checklist.

Statistics

For all statistical analyses, confirm that the following items are present in the figure legend, table legend, main text, or Methods section.

n/a	Confirmed	
☐	☒	The exact sample size (n) for each experimental group/condition, given as a discrete number and unit of measurement
☐	☒	A statement on whether measurements were taken from distinct samples or whether the same sample was measured repeatedly
☐	☒	The statistical test(s) used AND whether they are one- or two-sided<br>*Only common tests should be described solely by name; describe more complex techniques in the Methods section.*
☐	☒	A description of all covariates tested
☐	☒	A description of any assumptions or corrections, such as tests of normality and adjustment for multiple comparisons
☐	☒	A full description of the statistical parameters including central tendency (e.g. means) or other basic estimates (e.g. regression coefficient) AND variation (e.g. standard deviation) or associated estimates of uncertainty (e.g. confidence intervals)
☒	☐	For null hypothesis testing, the test statistic (e.g. F, t, r) with confidence intervals, effect sizes, degrees of freedom and P value noted<br>*Give P values as exact values whenever suitable.*
☒	☐	For Bayesian analysis, information on the choice of priors and Markov chain Monte Carlo settings
☒	☐	For hierarchical and complex designs, identification of the appropriate level for tests and full reporting of outcomes
☒	☐	Estimates of effect sizes (e.g. Cohen's d, Pearson's r), indicating how they were calculated

Our web collection on statistics for biologists contains articles on many of the points above.

Software and code

Policy information about availability of computer code

Data collection	All data pertaining to clinical outcomes and TP53 mutational status was collected in Microsoft Excel.
Data analysis	Missense TP53 mutations were analyzed using the Evolutionary Action Scoring (EAS) System: http://eaction.lichtargelab.org/<br>All analyses were performed and survival plots were created using R version 4.1.0.

For manuscripts utilizing custom algorithms or software that are central to the research but not yet described in published literature, software must be made available to editors and reviewers. We strongly encourage code deposition in a community repository (e.g. GitHub). See the Nature Portfolio guidelines for submitting code & software for further information.

Data

Policy information about availability of data

All manuscripts must include a data availability statement. This statement should provide the following information, where applicable:

- Accession codes, unique identifiers, or web links for publicly available datasets
- A description of any restrictions on data availability
- For clinical datasets or third party data, please ensure that the statement adheres to our policy

The data that support the findings are not publicly available, but may be made available upon reasonable request from the corresponding author, Dr. Zahi Mitri.

Field-specific reporting

Please select the one below that is the best fit for your research. If you are not sure, read the appropriate sections before making your selection.

☒ Life sciences ☐ Behavioural & social sciences ☐ Ecological, evolutionary & environmental sciences

For a reference copy of the document with all sections, see nature.com/documents/nr-reporting-summary-flat.pdf

Life sciences study design

All studies must disclose on these points even when the disclosure is negative.

Sample size	No sample size power calculations were used for this study. The results presented in this manuscript are a summary of clinical outcomes from patients that consented to participate in a phase II
Data exclusions	No data was excluded from the analysis.
Replication	No replications. This is a summary of clinical outcomes.
Randomization	No randomization was implemented. This was a single-institution, observational study that prospectively followed a cohort of patients with primary triple negative breast cancer.
Blinding	No blinding was employed.

Reporting for specific materials, systems and methods

We require information from authors about some types of materials, experimental systems and methods used in many studies. Here, indicate whether each material, system or method listed is relevant to your study. If you are not sure if a list item applies to your research, read the appropriate section before selecting a response.

Materials & experimental systems

n/a	Involved in the study	
☒	☐	Antibodies
☒	☐	Eukaryotic cell lines
☒	☐	Palaeontology and archaeology
☒	☐	Animals and other organisms
☐	☒	Human research participants
☒	☐	Clinical data
☒	☐	Dual use research of concern

Methods

n/a	Involved in the study	
☒	☐	ChIP-seq
☒	☐	Flow cytometry
☒	☐	MRI-based neuroimaging

Human research participants

Policy information about studies involving human research participants

Population characteristics	This manuscript reports on a study sub-population derived from patients that had consented to participate in prospective registry study to determine the feasibility of implementation of molecular (genomic) predictive testing for patients with localized (stage I-III), HER2 negative, invasive carcinoma of the breast who are candidates for either adjuvant or neoadjuvant treatment of their breast cancer (MDACC IRB#2011-0007). The study population reported in this manuscript focused solely on those study patients that were diagnosed with primary triple negative breast cancer between 2011 and 2015, and had their disease managed per standard of care.
Recruitment	Participation in this study was offered to new patients at the surgery and breast imaging clinics at MDACC.
Ethics oversight	Ethics oversight was provided by MDACC IRB

Note that full information on the approval of the study protocol must also be provided in the manuscript.
