# Supplementary material for: Impact of TP53 mutations in Triple Negative Breast Cancer
Source: NPJ Precis Oncol. 2022 Sep 9;6:64. doi: 10.1038/s41698-022-00303-6 (PMC9463132; doi:10.1038/s41698-022-00303-6)
Supplement: Supplementary file 2 — Informed Consent [file 41698_2022_303_MOESM2_ESM.pdf]

THE UNIVERSITY OF TEXAS
MD Anderson ~~Cancer~~ Center

Informed Consent

Please Do Not Use for Patient Consent

Go to the PDOL Homepage to access the Informed Consent Printer Database

INFORMED CONSENT/AUTHORIZATION FOR PARTICIPATION IN RESEARCH WITH OPTIONAL PROCEDURES

Feasibility, Validation and Implementation of Genomic Testing for Chemotherapy and Endocrine Sensitivity of HER2 Negative Primary Invasive Breast Cancer (Clinical Stage I to III)
2011-0007

Subtitle: Main Study Consent

Study Chair: Stacy Moulder

1. __________________________ __________________________

Participant's Name | Medical Record Number

You are being asked to take part in this clinical research study at The University of Texas MD Anderson Cancer Center ("MD Anderson"). This consent and authorization form explains why this research study is being done and what your role will be if you choose to take part. This form also describes the possible risks connected with being in this study. After reviewing this information with the person responsible for your enrollment, you should know enough to be able to make an informed decision on whether you want to take part in the study.

You are being asked to take part in this study because you have early stage breast cancer or a suspicious abnormality in the breast that is thought to be breast cancer.

2. PURPOSE OF STUDY

The goal of this research study is find out if researchers can use genetic testing on tumor samples to predict if tumors will respond to breast cancer treatments. The

tumor sample will be tested to learn if certain genes are activated (turned on) in the tumor. Researchers hope that the activation of these genes may predict if the tumor will be sensitive or resistant to routine breast cancer treatments, such as chemotherapy or hormonal therapy.

3. DESCRIPTION OF STUDY

This study will involve performing a test on a sample of tumor. If you agree to take part in this study, you will have a tumor biopsy before you receive any drugs to treat breast cancer. The sample of the tumor will be will be taken at one of the following times:

- At the time of a planned biopsy to learn if you have breast cancer.
- At the time of planned surgery to remove a known breast cancer.
- If the breast cancer has been previously biopsied and your doctor plans to give you breast cancer treatments before surgery, you will have a biopsy before starting these drugs.

The amount of tumor collected for this study will be about the size of the tip of a pencil.
If you are going to have surgery to remove the breast cancer before receiving breast cancer treatments, a small piece of the tumor will be removed and sent for testing. If you are going to have a planned needle biopsy, an extra core sample and/or fine needle sample will be taken. If you are going to have a research biopsy, an ultrasound or mammogram will be used to find the tumor and a needle will be inserted into the tumor to collect a piece of tissue.

After the genetic testing is complete, researchers will use the results to learn how well this test is able to give results that can be used to predict response to breast cancer drugs and therapy.

Test Results
Because this is an investigational test, neither you or your doctor will be told the results of this test. This test will also not be used to guide your doctor's choice of therapy. This study will only look at how well this test was able to predict the response of the tumor to breast cancer treatment.

For 5 years, the study staff will review your medical record to see how you are responding to any breast cancer therapies that you receive. If you are treated outside MD Anderson, you will be called and asked how you are doing. This phone call should take about 5 minutes.

To protect your privacy, all information stored as part of this study will be kept secure and confidential.

Length of Study

You will be off study after the 5 years of follow-up.

This is an investigational study. The test that will be performed on your breast cancer tumor sample is an investigational test.

If you need to have a biopsy for this study, it will be performed at no cost to you. If tissue is being collected during a regularly scheduled biopsy, you and/or your insurance provider will be responsible for the cost of the biopsy. The testing performed on your tumor sample will be performed at no cost to you.

Up to 1100 patients will take part in this study. All will be enrolled at MD Anderson.

4. RISKS, SIDE EFFECTS, AND DISCOMFORTS TO PARTICIPANTS

Having **biopsies** performed may cause pain, bruising, bleeding, redness, low blood pressure, swelling, and/or infection at the site of the biopsies. An allergic reaction to the anesthetic may occur. A scar may form at the biopsy site.

This study may involve unpredictable risks to the participants.

5. POTENTIAL BENEFITS

The results of the test performed on your tumor sample will not be made known to you or your doctor. Future patients may benefit from what is learned. There may be no benefits for you in this study.

6. ALTERNATIVE PROCEDURES OR TREATMENTS

You may choose not to take part in this study. In all cases, you will receive appropriate medical care.

OPTIONAL PROCEDURES FOR THE STUDY

Optional Procedure #1: If you agree, you will have an additional tumor sample collected during the biopsy planned for this study. No additional biopsy will be required, we will only collect another sample at the same time point.

This sample will be used for research and placed into mice where the cancer cells can grow. By doing this, researchers are able to treat the mice to determine the effect of drugs against cancer cells. Not all human cancers will grow as tumors in mice and the results of these tests will not be used to guide your treatment

Optional Procedure #2: If you agree, any tissue left over after the testing will be stored in a research tissue bank at MD Anderson for use in future research related to cancer.

Before this tissue can be used for future research, the people doing the research must get separate approval from the Institutional review board of MD Anderson (IRB), and your consent and authorization, or a waiver of consent and authorization from the IRB. The IRB is a committee made up of doctors, researchers, and members of the community. The IRB is responsible for protecting the participants involved in research studies and making sure all research is done in a safe and ethical manner. All research done at MD Anderson, including research involving your tissue from this bank, must first be approved by the IRB.

Your samples will be given a code number. No identifying information will be directly linked to your samples. Only the researcher in charge of the bank will have access to the code numbers and be able to link the samples to you. This is to allow medical data related to the samples to be updated as needed. Other researchers using your samples will not be able to link this data to you.

There are no benefits to you for taking part in the optional procedures. Future patients may benefit from what is learned. You may stop taking part at any time. There will be no cost to you for taking part in the optional procedure.

You do not have to agree to take part in the optional procedures in order to be enrolled in this study.

Optional Procedure Risks:

Having **biopsies** performed may cause pain, bruising, bleeding, redness, low blood pressure, swelling, and/or infection at the site of the biopsies. An allergic reaction to the anesthetic may occur. A scar may form at the biopsy site.

MD Anderson and others can learn about cancer and other diseases from your **banked tissue**. In the future, people who may do research with these samples may need to know more information about your health. This information may be collected from your medical record. MD Anderson will make reasonable efforts to

preserve your privacy, but cannot guarantee complete privacy. Sometimes your samples may be used for genetic research about diseases that are passed on in families.

Genetic research may result in the development of beneficial treatments, devices, new drugs, or patentable procedures. There are no plans to provide you compensation from such developments. MD Anderson will not be able to give you, your family, or your doctor the reports about the research done with these samples, and these reports will not be put in your medical record. If this information were released, it could be misused. Such misuse could be distressing, and it could cause you or your family members to have difficulty obtaining insurance coverage and/or a job.

If you withdraw your consent to the storage of leftover tissue in the tissue bank, then the leftover materials will no longer be collected for storage. Any of your tissue that remains in the tissue bank will no longer be used for research and will be removed from the tissue bank and destroyed.

However, if any of your de-identified tissue was already released for research purposes before you withdrew consent, MD Anderson will not be able to destroy it.

CONSENT/PERMISSION/AUTHORIZATION FOR OPTIONAL PROCEDURES

Circle your choice of "yes" or "no" for each of the following optional procedures:

Optional Procedure #1: Do you agree to allow one additional tumor sample to be collected for cancer research in mice?

YES **NO**

Optional Procedure #2: Do you agree to allow leftover tissue to be stored in a research tissue bank at MD Anderson for use in future research related to cancer?

YES **NO**

Additional Information

7. You may ask the study chair any questions you have about this study. You may contact the study chair, Dr. Stacy Moulder, at 713-792-2817. You may also contact the Chair of MD Anderson's Institutional Review Board (IRB - a committee that reviews research studies) at 713-792-2933 with any questions that have to do with this study or your rights as a study participant.

8. Your participation in this research study is strictly voluntary. You may choose not to take part in this study without any penalty or loss of benefits to which you are otherwise entitled. You may also withdraw from participation in this study at any time without any penalty or loss of benefits. If you decide you want to stop taking part in the study, it is recommended for your safety that you first talk to your doctor. If you withdraw from this study, you can still choose to be treated at MD Anderson.

9. This study or your participation in it may be changed or stopped at any time by the study chair, Komen Foundation, the U.S. Food and Drug Administration (FDA), the Office for Human Research Protections (OHRP - a regulatory agency that oversees research in humans), or the IRB of MD Anderson.

10. You will be informed of any new findings that might affect your willingness to continue taking part in the study.

11. MD Anderson may benefit from your participation and/or what is learned in this study.

    Dr. W. F. Symmans (Collaborator) has an equity or stock option interest in Nuvera Biosciences, Incorporated, a company that has developed microarray-based predictors (a type of genetic testing). The company is not supporting this study, but the microarray-based predictors will be used in this study.

    The University of Texas MD Anderson has an equity or stock option interest in Nuvera Biosciences, Incorporated, a company that has developed microarray-based predictors. The company is not supporting this study, but the microarray-based predictors will be used in this study.

12. This study is supported by: Komen Foundation.

STUDY COSTS AND COMPENSATION

If you suffer injury as a direct result of taking part in this study, MD Anderson health providers will provide medical care. However, this medical care will be billed to your insurance provider or you in the ordinary manner. You will not be reimbursed for expenses or compensated financially by MD Anderson or Komen Foundation for this injury. You may also contact the Chair of MD Anderson's IRB at 713-792-2933 with questions about study-related injuries. By signing this consent form, you are not giving up any of your legal rights.

Certain tests, procedures, and/or drugs that you may receive as part of this study may be without cost to you because they are for research purposes only. However, your insurance provider and/or you may be financially responsible for the cost of care and treatment of any complications resulting from the research tests, procedures, and/or drugs, including hospitalization, nausea, vomiting, low blood cell counts, and dehydration. Standard medical care that you receive under this research study will be billed to your insurance provider and/or you in the ordinary manner. Before taking part in this study, you may ask about which parts of the research-related care may be provided without charge, which costs your insurance provider may pay for, and which costs may be your responsibility. You may ask that a financial counselor be made available to you to talk about the costs of this study.

There are no plans to compensate you for any patents or discoveries that may result from your participation in this research.

You will receive no compensation for taking part in this study.

Authorization for Use and Disclosure of Protected Health Information:

A. During the course of this study, the research team at MD Anderson will be collecting and using your protected health information. This information may include personal identifying information about you (such as your name, race, date of birth, gender, city, and zip code), your medical history, study schedule, and the results of any of your tests, therapies, and/or procedures. The purpose of collecting and sharing this information is to learn about how the study procedures may affect the disease and any study-related side effects. Your doctor and the research team may share your study information with the parties named in Section D below.

All testing for this study will be performed in the CLIA certified Molecular Diagnostics lab at MD Anderson.

B. Signing this consent and authorization form is optional. However, if you refuse to provide your authorization to use and disclose your protected health information for this study, you will not be able to participate in this research project.

C. MD Anderson will take appropriate steps to keep your protected health information private when possible, and it will be protected according to state and federal law. However, there is no guarantee that your information will remain confidential, and it may be re-disclosed at some point. Federal agencies (such as the FDA, OHRP, or National Cancer Institute [NCI]), Komen Foundation, and the IRB of MD Anderson might view or receive your record in order to collect data and/or meet legal, ethical, research, and safety-related obligations. In some situations, the FDA could be required to reveal the names of participants.

D. Your protected health information may be shared with the following parties:

- Komen Foundation (and/or any future sponsors of the study)
- Federal agencies that require reporting of clinical study data (such as the FDA, NCI, and OHRP)
- The IRB of MD Anderson
- Officials of MD Anderson
- Study monitors who verify the accuracy of the information
- Individuals who put all the study information together in report form

E. Normally you have a right to access your medical record. However, in order to preserve the integrity of this research study, you will not be permitted to have access to certain portions of your medical record while the study is ongoing.

F. There is no expiration date for the use of your protected health information. You may withdraw your authorization to share your protected health information at any time in writing. Instructions on how to do this can be found in the MD Anderson Notice of Privacy Practices (NPP). You may contact the IRB Staff at 713-792-2933 with questions about how to find the NPP. If you withdraw your authorization, you will be removed from the study, and the study chair and staff will no longer use or disclose your protected health information in connection with this study, unless the study chair or staff needs to use or disclose some of your research-related personal health information to preserve the scientific value of the study. Data collected about you up to the time you withdrew will be used and included in the data analysis. The parties listed in Section D above may use and disclose any study data that were collected before you canceled your authorization.

G. A description of this clinical trial will be available on http://www.ClinicalTrials.gov, as required by U.S. Law. This Web site will not include information that can identify you. At most, the Web site will include a summary of the results. You can search this Web site at any time.

Please Do Not Use for Patient Consent
Go to the PDOL Homepage to access the
Informed Consent Printer Database

CONSENT/AUTHORIZATION

I understand the information in this consent form. I have had a chance to read the consent form for this study, or have had it read to me. I have had a chance to think about it, ask questions, and talk about it with others as needed. I give the study chair permission to enroll me on this study. By signing this consent form, I am not giving up any of my legal rights. I will be given a signed copy of this consent document.

SAMPLE -- NOT FOR USE IN CONSENTING PATIENTS
SIGNATURE OF PARTICIPANT   DATE

LEGALLY AUTHORIZED REPRESENTATIVE (LAR)

The following signature line should only be filled out when the participant does not have the capacity to legally consent to take part in the study and/or sign this document on his or her own behalf.

SAMPLE -- NOT FOR USE IN CONSENTING PATIENTS
SIGNATURE OF LAR   DATE

SAMPLE -- NOT FOR USE IN CONSENTING PATIENTS
RELATIONSHIP TO PARTICIPANT

WITNESS TO CONSENT

I was present during the explanation of the research to be performed under Protocol **2011-0007**.

SAMPLE -- NOT FOR USE IN CONSENTING PATIENTS
SIGNATURE OF WITNESS TO THE VERBAL CONSENT PRESENTATION (OTHER THAN PHYSICIAN OR STUDY CHAIR)   DATE

A witness signature is only required for vulnerable adult participants. If witnessing the assent of a pediatric participant, leave this line blank and sign on the witness to assent page instead.

PERSON OBTAINING CONSENT

NOT FOR USE IN CONSENTING PATIENTS

I have discussed this clinical research study with the participant and/or his or her authorized representative, using language that is understandable and appropriate. I believe that I have fully informed this participant of the nature of this study and its possible benefits and risks and that the participant understood this explanation.

SAMPLE -- NOT FOR USE IN CONSENTING PATIENTS

SIGNATURE OF STUDY CHAIR
OR PERSON AUTHORIZED TO OBTAIN CONSENT

DATE

TRANSLATOR

I have translated the above informed consent as written (without additions or subtractions) into ______________________________ and assisted the people
(Name of Language)
obtaining and providing consent by translating all questions and responses during the consent process for this participant.

SAMPLE -- NOT FOR USE IN CONSENTING PATIENTS

NAME OF TRANSLATOR	SIGNATURE OF TRANSLATOR	DATE

☐ Please check here if the translator was a member of the research team. (If checked, a witness, other than the translator, must sign the witness line below.)

SAMPLE -- NOT FOR USE IN CONSENTING PATIENTS

SIGNATURE OF WITNESS TO THE VERBAL TRANSLATION (OTHER THAN TRANSLATOR, PARENT/GUARDIAN, OR STUDY CHAIR)	DATE
