# Supplementary material for: Impact of TP53 mutations in Triple Negative Breast Cancer
Source: NPJ Precis Oncol. 2022 Sep 9;6:64. doi: 10.1038/s41698-022-00303-6 (PMC9463132; doi:10.1038/s41698-022-00303-6)
Supplement: Supplementary file 3 — Supplementary Material [file 41698_2022_303_MOESM3_ESM.pdf]

Supplemental Table 1. Summary of TP53 Mutations in study cohort. Bolded cases represent TP53 mutations in the EAp53 1-69 group. For the 2 patients with >1 TP53 mutation (1 patient had 2, 1 patient had 3), the mutation with the highest Evolutionary Action Score was chosen for analysis and reported here.

Sample ID	TP53 mutation	Mut. type	Chromosome	Mut. location	Mut. function*
MD01	A159P	Missense	17	DNA binding	Non-disruptive
**MD02**	**A161T**	**Missense**	**17**	**DNA binding**	**Non-disruptive**
MD03	C135Y	Missense	17	DNA binding	Non-disruptive
MD04	C176R	Missense	17	DNA binding	Disruptive
MD05	C275F	Missense	17	DNA binding	Non-disruptive
MD06	C275Y	Missense	17	DNA binding	Non-disruptive
MD07	D281G	Missense	17	DNA binding	Non-disruptive
MD08	E258Q	Missense	17	DNA binding	Non-disruptive
MD09	E286K	Missense	17	DNA binding	Non-disruptive
MD10	E339*	Nonsense	17	other residue	Disruptive
MD11	E56*	Nonsense	17	other residue	Disruptive
**MD12**	**F270L**	**Missense**	**17**	**DNA binding**	**Non-disruptive**
MD13	G199*	Nonsense	17	DNA binding	Disruptive
MD14	G245S	Missense	17	DNA binding	Disruptive
MD15	G266E	Missense	17	DNA binding	Non-disruptive
MD16	G266R	Missense	17	DNA binding	Non-disruptive
MD17	H193R	Missense	17	DNA binding	Non-disruptive
MD18	H193R	Missense	17	DNA binding	Non-disruptive
**MD19**	**K132R**	**Missense**	**17**	**DNA binding**	**Non-disruptive**
MD20	L194R	Missense	17	DNA binding	Disruptive
MD21	L194R	Missense	17	DNA binding	Disruptive
**MD22**	**L308P**	**Missense**	**17**	**other residue**	**Non-disruptive**
MD23	L348*	Nonsense	17	other residue	Disruptive
MD24	P278S	Missense	17	DNA binding	Non-disruptive
MD25	Q317*	Nonsense	17	other residue	Disruptive
MD26	R175H	Missense	17	DNA binding	Non-disruptive
MD27	R175H	Missense	17	DNA binding	Non-disruptive
MD28	R175H	Missense	17	DNA binding	Non-disruptive
MD29	R175H	Missense	17	DNA binding	Non-disruptive
MD30	R175H	Missense	17	DNA binding	Non-disruptive
MD31	R213*	Nonsense	17	DNA binding	Disruptive
MD32	R213*	Nonsense	17	DNA binding	Disruptive
MD33	R213*	Nonsense	17	DNA binding	Disruptive
MD34	R213*	Nonsense	17	DNA binding	Disruptive
MD35	R248Q	Missense	17	DNA binding	Disruptive
MD36	R248W	Missense	17	DNA binding	Disruptive
MD37	R248W	Missense	17	DNA binding	Disruptive
MD38	R273C	Missense	17	DNA binding	Non-disruptive
MD39	R273C	Missense	17	DNA binding	Non-disruptive
MD40	R273G	Missense	17	DNA binding	Non-disruptive
MD41	R280K	Missense	17	DNA binding	Non-disruptive
**MD42**	**R337L**	**Missense**	**17**	**other residue**	**Non-disruptive**
MD43	R342*	Nonsense	17	other residue	Disruptive
MD44	S166*	Nonsense	17	DNA binding	Disruptive
MD45	V173L	Missense	17	DNA binding	Non-disruptive

Supplemental Table 1. Summary of TP53 Mutations in study cohort. Bolded cases represent TP53 mutations in the EAp53 1-69 group. For the 2 patients with >1 TP53 mutation (1 patient had 2, 1 patient had 3), the mutation with the highest Evolutionary Action Score was chosen for analysis and reported here.

Sample ID	TP53 mutation	Mut. type	Chromosome	Mut. location	Mut. function*
MD46	V216E	Missense	17	DNA binding	Non-disruptive
MD47	V216M	Missense	17	DNA binding	Non-disruptive
MD48	V217G	Missense	17	DNA binding	Non-disruptive
**MD49**	**V274A**	**Missense**	**17**	**DNA binding**	**Non-disruptive**
MD50	Y107*	Nonsense	17	DNA binding	Disruptive
MD51	Y126C	Missense	17	DNA binding	Non-disruptive
MD52	Y126*	Nonsense	17	DNA binding	Disruptive
MD53	Y220C	Missense	17	DNA binding	Non-disruptive
MD54	Y220C	Missense	17	DNA binding	Non-disruptive
MD55	Y220N	Missense	17	DNA binding	Non-disruptive

* Disruptive and non-disruptive classification of TP53 mutation based on predicted degree of disturbance of the p53 protein structure and function as reported by Poeta et al[1] and Molina-Vila et al[2].

Supplemental Table 2. Post-diagnosis RFS and OS univariable Cox model results

		3-year Recurrence-free survival rate			3-year Overall survival rate		
**Variables**	**N**	**% (95% CI)**	**HR (95% CI)**	***p***	**% (95% CI)**	**HR (95% CI)**	***p***
Age at diagnosis*			1.24 (1.03 - 1.49)	0.023		1.24 (1.01 - 1.51)	0.036
Age (binary)							
<50 years	30	89.8 (71.5 - 96.6)			93.1 (75.1 - 98.2)		
≥50 years	66	73.8 (61.3 - 82.9)	2.70 (0.93 - 7.85)	0.067	81.4 (69.5 - 89.0)	2.88 (0.85 - 9.78)	0.09
Race							
white	59	77.3 (64.1 - 86.1)			85.9 (73.7 - 92.7)		
non-white	36	83.3 (66.6 - 92.1)	0.70 (0.30 - 1.63)	0.41	86.0 (69.6 - 93.9)	0.81 (0.32 - 2.02)	0.645
Laterality							
Left	43	81.4 (66.2 - 90.2)			85.8 (71.1 - 93.4)		
Right	53	76.6 (62.5 - 86.0)	1.36 (0.62 - 3.00)	0.443	84.3 (71.1 - 91.8)	1.08 (0.46 - 2.57)	0.861
Tumor volume (cm^3)			1.00 (1.00 - 1.00)	0.837		1.00 (1.00 - 1.00)	0.843
Clinical T stage							
T1	17	94.1 (65.0 - 99.1)			94.1 (65.0 - 99.1)		
T2	66	78.5 (66.4 - 86.6)	5.68 (0.76 - 42.34)	0.09	86.2 (75.1 - 92.5)	4.23 (0.56 - 31.86)	0.162
T3/T4	13	58.7 (27.4 - 80.4)	8.74 (1.02 - 74.99)	0.048	64.8 (31.0 - 85.2)	7.43 (0.83 - 66.59)	0.073
Clinical nodal status							
Negative	55	92.6 (81.5 - 97.2)			96.3 (86.0 - 99.1)		
Positive	41	**60.3 (43.5 - 73.5)**	**3.77 (1.63 - 8.68)**	**0.002**	**69.5 (52.6 - 81.4)**	**4.28 (1.66 - 11.05)**	**0.003**
Clinical N stage							
N0	55	92.6 (81.5 - 97.2)			96.3 (86.0 - 99.1)		
N1	22	**63.6 (40.3 - 79.9)**	**3.17 (1.22 - 8.24)**	**0.018**	**68.2 (44.6 - 83.4)**	**4.39 (1.56 - 12.34)**	**0.005**
N2/N3	19	**56.1 (31.1 - 75.2)**	**4.66 (1.78 - 12.15)**	**0.002**	**70.8 (43.2 - 86.8)**	**4.12 (1.32 - 12.86)**	**0.015**
Clinical TNM stage							
I	13	100.0 (100 - 100)	Unstable HR		100.0 (100 - 100)	Unstable HR	
II	59	82.8 (70 - 90)			87.9 (76 - 94)		
III/IV	24	57.0 (35 - 74)			68.7 (45 - 84)		
Tumor grade							
II	15	79.4 (48.8 - 92.9)			78.6 (47.2 - 92.5)		
III	79	78.2 (67.3 - 85.8)	1.51 (0.45 - 5.04)	0.504	85.8 (75.8 - 91.9)	1.15 (0.34 - 3.91)	0.824
Histology							
IDC	90	78.7 (68.7 - 85.9)			85.3 (76.1 - 91.2)		
Other	6	80.0 (20.4 - 96.9)	0.63 (0.08 - 4.65)	0.649	80.0 (20.4 - 96.9)	0.81 (0.11 - 6.06)	0.839

Supplemental Table 2. Post-diagnosis RFS and OS univariable Cox model results

		3-year Recurrence-free survival rate			3-year Overall survival rate		
**Variables**	**N**	**% (95% CI)**	**HR (95% CI)**	***p***	**% (95% CI)**	**HR (95% CI)**	***p***
TP53 expression			1.01 (0.98 - 1.03)	0.56		1.02 (0.99 - 1.04)	0.202
TP53 mutation							
WT	41	80.0 (64.0 - 89.5)			90.0 (75.5 - 96.1)		
Mutated	55	77.9 (64.4 - 86.8)	1.15 (0.52 - 2.54)	0.726	81.3 (68.0 - 89.5)	1.54 (0.62 - 3.82)	0.351
TP53 (DNA-binding)							
WT	41	80.0 (64.0 - 89.5)			90.0 (75.5 - 96.1)		
DNA binding	48	80.9 (66.5 - 89.6)	0.97 (0.42 - 2.24)	0.941	84.9 (70.8 - 92.5)	1.23 (0.47 - 3.23)	0.677
Other residue	7	57.1 (17.2 - 83.7)	2.66 (0.83 - 8.50)	0.099	**57.1 (17.2 - 83.7)**	**4.25 (1.24 - 14.57)**	**0.021**
TP53 (Disruptive)							
WT	41	80.0 (64.0 - 89.5)			90.0 (75.5 - 96.1)		
Non-disruptive	35	76.7 (58.7 - 87.6)	1.27 (0.54 - 2.99)	0.584	82.0 (64.2 - 91.5)	1.58 (0.59 - 4.25)	0.363
Disruptive	20	80.0 (55.1 - 92.0)	0.96 (0.33 - 2.80)	0.935	80.0 (55.1 - 92.0)	1.47 (0.47 - 4.64)	0.509
EASp53**							
0	41	80.0 (64.0 - 89.5)			90.0 (75.5 - 96.1)		
1 - 99	42	78.2 (62.3 - 88.0)	1.15 (0.49 - 2.65)	0.752	82.6 (67.0 - 91.3)	1.46 (0.56 - 3.84)	0.441
100	13	76.9 (44.2 - 91.9)	1.17 (0.37 - 3.75)	0.787	76.9 (44.2 - 91.9)	1.78 (0.52 - 6.11)	0.358
EASp53 optimal							
0	41	80.0 (64.0 - 89.5)			90.0 (75.5 - 96.1)		
1 - 69	6	**50.0 (11.1 - 80.4)**	**4.17 (1.41 - 12.31)**	**0.01**	**66.7 (19.5 - 90.4)**	**5.33 (1.55 - 18.39)**	**0.008**
70 - 99	36	82.9 (65.9 - 91.9)	0.76 (0.29 - 1.99)	0.571	85.6 (68.8 - 93.8)	0.99 (0.33 - 2.94)	0.983
100	13	76.9 (44.2 - 91.9)	1.18 (0.37 - 3.78)	0.776	76.9 (44.2 - 91.9)	1.80 (0.52 - 6.16)	0.35

**Continuous variable so no group-specific 3-year survival estimates. For each categorical variable, the top group listed is the reference level (i.e., denominator) for the Hazard Ratio.*

Supplemental Table 3. Post-surgery RFS and OS univariable Cox model results

		3-year Recurrence-free survival rate			3-year Overall survival rate		
**Variables**	**N**	**% (95% CI)**	**HR (95% CI)**	***p***	**% (95% CI)**	**HR (95% CI)**	***p***
Dx to NACT, months*			0.96 (0.59 - 1.57)	0.876	-	1.07 (0.66 - 1.72)	0.789
Taxane therapy							
No	2	100.0 (NA - NA)			100.0 (NA - NA)		
Yes	65	76.9 (64.7 - 85.4)	0.53 (0.07 - 3.99)	0.537	81.3 (69.5 - 89.0)	0.47 (0.06 - 3.59)	0.47
Anthracycline tx							
No	4	75.0 (12.8 - 96.1)			75.0 (12.8 - 96.1)		
Yes	63	77.8 (65.4 - 86.2)	0.50 (0.11 - 2.15)	0.349	82.3 (70.4 - 89.8)	0.43 (0.10 - 1.87)	0.259
Platinum therapy							
No	59	78.0 (65.1 - 86.6)			82.8 (70.4 - 90.4)		
Yes	8	75.0 (31.5 - 93.1)	0.95 (0.22 - 4.11)	0.947	75.0 (31.5 - 93.1)	1.30 (0.29 - 5.71)	0.731
Dx to Sx, months*			1.11 (0.73 - 1.68)	0.623		1.33 (0.85 - 2.07)	0.216
Path T stage							
T0/Tis	26	100.0 (NA - NA)	Unstable HR		100.0 (NA - NA)	Unstable HR	
T1	22	81.8 (58.5 - 92.8)			86.4 (63.4 - 95.4)		
T2	10	60.0 (25.3 - 82.7)			70.0 (32.9 - 89.2)		
T3	9	22.2 (3.4 - 51.3)			27.8 (4.4 - 59.1)		
Path N stage							
N0	49	93.9 (82.2 - 98.0)			93.9 (82.2 - 98.0)		
N1	12	**41.7 (15.2 - 66.5)**	**8.43 (2.99 - 23.83)**	**<0.001**	**64.2 (30.2 - 84.8)**	**7.07 (2.24 - 22.37)**	**0.001**
N2/N3	6	**16.7 (0.8 - 51.7)**	**19.76 (5.76 - 67.77)**	**<0.001**	**16.7 (0.8 - 51.7)**	**25.14 (6.93 - 91.22)**	**<0.001**
Path TNM stage							
0	24	100.0 (NA - NA)	Unstable HR		100.0 (NA - NA)	Unstable HR	
I	17	88.2 (60.6 - 96.9)			88.2 (60.6 - 96.9)		
II	17	70.6 (43.1 - 86.6)			82.4 (54.7 - 93.9)		
III	9	11.1 (0.6 - 38.8)			14.8 (0.8 - 46.8)		
pCR, after NACT							
Yes	24	100.0 (NA - NA)	Unstable HR		100.0 (NA - NA)	Unstable HR	
No	43	65.1 (49.0 - 77.3)			71.7 (55.6 - 82.8)		
RCB 0/1, after NACT							
0/1	29	96.6 (77.9 - 99.5)			96.6 (77.9 - 99.5)		
2/3	36	**61.1 (43.3 - 74.8)**	**20.1 (2.7 - 150.7)**	**0.003**	**69.0 (51.0 - 81.5)**	**15.8 (2.1 - 118.8)**	**0.007**

Supplemental Table 3. Post-surgery RFS and OS univariable Cox model results

		3-year Recurrence-free survival rate			3-year Overall survival rate		
**Variables**	**N**	**% (95% CI)**	**HR (95% CI)**	***p***	**% (95% CI)**	**HR (95% CI)**	***p***
RCB index, NACT							
0	24	100.0 (NA - NA)	Unstable HR		100.0 (NA - NA)	Unstable HR	
I	5	80.0 (20.4 - 96.9)			80.0 (20.4 - 96.9)		
II	25	80.0 (58.4 - 91.1)			84.0 (62.8 - 93.7)		
III	11	18.2 (2.9 - 44.2)			32.7 (8.3 - 60.6)		

**Continuous variable so no group-specific 3-year survival estimates. For each categorical variable, the top group listed is the reference level (i.e., denominator) for the Hazard Ratio.*

Supplemental Table 4. Recurrence-free survival Cox regression univariable model results for 43 patients that failed to achieve a pathological complete response after NACT.

Variable	N	3-year RFS rate (95% CI)	Hazard Ratio (95%CI)	p-value
**TP53 mutation**				
WT	18	77.8 (51.1 - 91.0)		
Mutated	25	56.0 (34.8 - 72.7)	2.14 (0.82 - 5.61)	0.12
**TP53 location**				
WT	18	77.8 (51.1 - 91.0)		
DNA binding	21	61.9 (38.1 - 78.8)	1.68 (0.61 - 4.63)	0.32
Other residue	4	**25.0 (0.9 - 66.5)**	**6.97 (1.93 - 25.10)**	**0.003**
**EAp53***				
0	18	77.8 (51.1 - 91.0)		
1 - 99	19	57.9 (33.2 - 76.3)	1.90 (0.69 - 5.25)	0.217
100	6	50.0 (11.1 - 80.4)	3.17 (0.88 - 11.38)	0.077
**EAp53 (RFS-optimal)**				
0	18	77.8 (51.1 - 91.0)		
1 - 69	6	50.0 (11.1 - 80.4)	3.31 (1.00 - 11.02)	0.051
70 - 99	13	61.5 (30.8 - 81.8)	1.34 (0.41 - 4.40)	0.63
100	6	50.0 (11.1 - 80.4)	3.14 (0.88 - 11.26)	0.079

Note: For each variable, the first category listed is the reference group (i.e., denominator) for the Hazard Ratio. Reported p-values are from a Wald test.

* TP53 status categorized as wild-type (EAp53 0), missense mutation (EAp53 1-99), and nonsense mutation (EAp53 100)

Supplemental Figure 1. Association of EAp53 score with clinical outcomes. (a) OS Kaplan–Meier curves by tumor TP53 mutation status and location (log rank test p = 0.034). **(b)** Line plot of the results of a RFS Cox model fit to a penalized spline basis of EAS for the 42 *TP53mis* patients, with a rug plot of the EAp53 distribution. **(c)** OS Kaplan–Meier curves by EAp53 category (log-rank test p = 0.015).

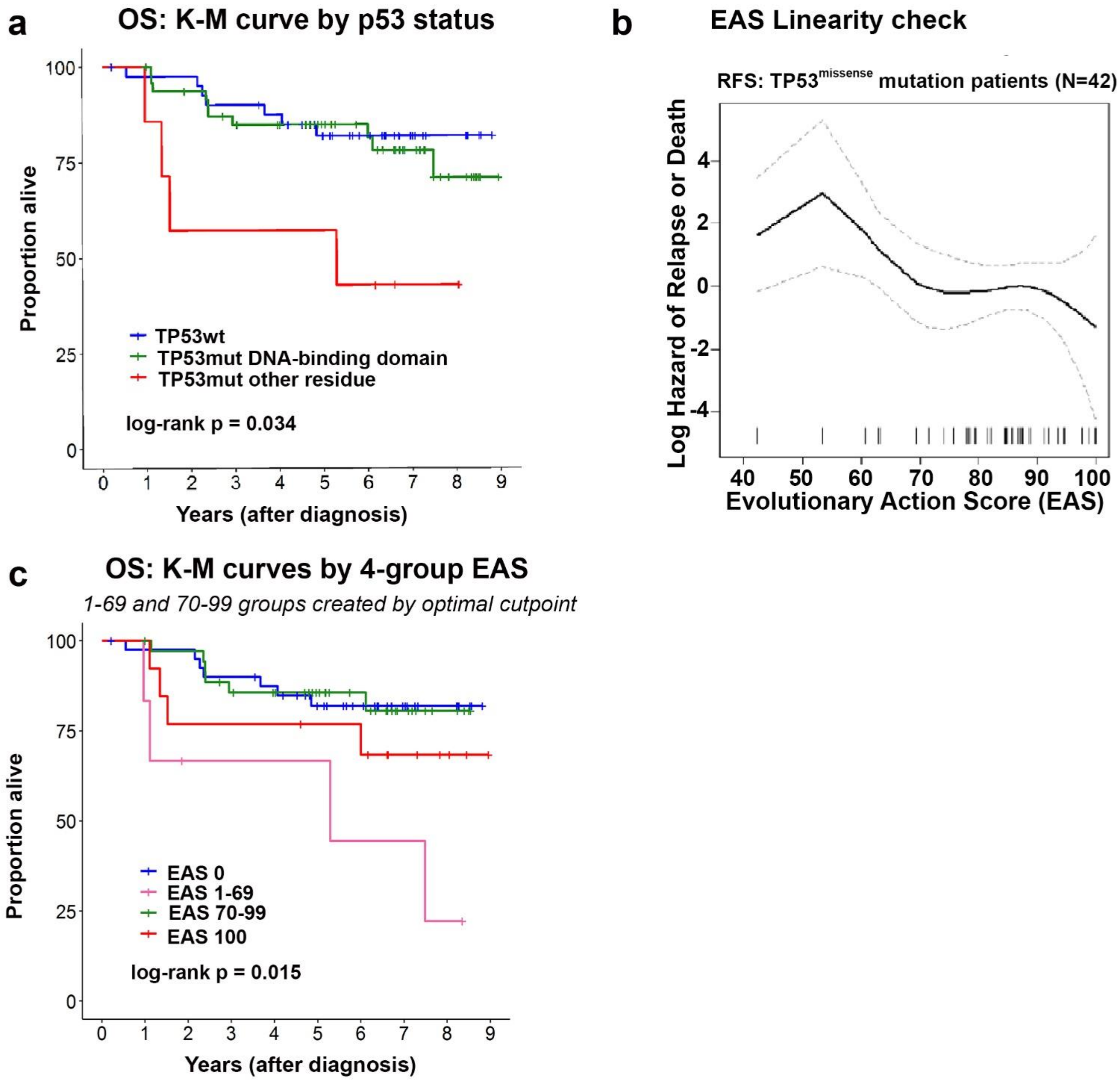

Supplemental References

1. Poeta, M.L., *et al.* TP53 Mutations and Survival in Squamous-Cell Carcinoma of the Head and Neck. *New England Journal of Medicine* **357**, 2552-2561 (2007).
2. Molina-Vila, M.A., *et al.* Nondisruptive p53 mutations are associated with shorter survival in patients with advanced non-small cell lung cancer. *Clin Cancer Res* **20**, 4647-4659 (2014).
